# Molecular Drivers of Cognitive Decline and Neuroprotection in Obstructive Sleep Apnea: A Narrative Review

**DOI:** 10.3390/antiox15091189

**Published:** 2026-09-17

**Authors:** Julia Jaromirska, Piotr Białasiewicz, Dominik Strzelecki, Agata Gabryelska

**Affiliations:** 1 Department of Sleep Medicine and Metabolic Disorders, Medical University of Lodz, 92-215 Lodz, Poland; julia.jaromirska@stud.umed.lodz.pl (J.J.); piotr.bialasiewicz@umed.lodz.pl (P.B.); 2 Department of Affective and Psychotic Disorders, Medical University of Lodz, 92-216 Lodz, Poland; dominik.strzelecki@umed.lodz.pl

**Keywords:** obstructive sleep apnea, neuroprotection, neurodegeneration, cognition

## Abstract

Obstructive sleep apnea is a common sleep disorder characterized by chronic intermittent hypoxia (CIH), sleep fragmentation, and upper-airway collapse. Beyond respiratory issues, OSA significantly impacts the central nervous system, increasing the risk for mild cognitive impairment and dementia. This review synthesizes the dual nature of CIH-induced molecular cascades, describing pathways driving neurodegeneration, such as SUMOylation dysregulation, toll-like receptor signaling, and inflammasome overactivation (largely characterized in experimental and animal CIH models), and those activating endogenous neuroprotective adaptations. Physiological defense mechanisms, such as the upregulation of erythropoietin, shifts toward anaerobic glycolysis, and the activation of neurotrophic factors, may act to mitigate neuronal injury. Rather than acting as independent processes, these molecular responses form a dynamic balance between neuronal injury and endogenous neuroprotection. Here, we introduce the concept of ‘neuroprotective reserve’ as a novel, hypothesis-generating framework to describe this intrinsic buffering capacity against hypoxic stress. Understanding and clinically validating these pathways is critical for developing early diagnostic biomarkers and targeted adjunctive interventions that complement standard therapy to prevent irreversible neurocognitive decline.

## 1. Introduction

Obstructive sleep apnea (OSA) is increasingly prevalent, shaped by a multifaceted interplay of obesity, advancing age, male sex, craniofacial anatomy, neuromuscular tone, and evolving diagnostic criteria. Following repeated collapse of the upper airways, patients with OSA present not only with chronic intermittent hypoxia (CIH), but also with sleep fragmentation and excessive daytime sleepiness [1,2]. It is further associated with neurological function [3], cardiovascular diseases [4], psychiatric well-being [5], and endocrine status [6]. Due to multiple complications caused by the disorder, patients with OSA need comprehensive care, which exceeds addressing only the baseline disorder. Apart from obesity [7] and age, risk factors for OSA include anatomical features such as enlarged tonsils, a recessed jaw, or nasal obstruction; several genes have been identified as associated with the disorder [8]. In addition, the course of the disease differs depending on the patient’s sex—females tend to have less severe and less position-dependent OSA, present with lower prevalence, but exhibit more concomitant symptoms, such as insomnia, restless leg syndrome, depression, nightmares, or hallucinations [9]. Overnight polysomnography (PSG) serves as the primary objective diagnostic tool for OSA, while continuous positive airway pressure (CPAP) represents the established first-line therapy [10]; however, therapeutic decisions are individually tailored by considering disease severity, anatomical features, clinical phenotypes, comorbidities, and patient preference. Late or incorrect diagnosis can increase the likelihood of developing concomitant ailments [11].

Due to CIH and associated oxidative stress presence, especially in the central nervous system, which plays a detrimental role in deterioration of cognitive functions, OSA patients are more prone to develop in particular mild cognitive impairment and further dementia [12], as the hippocampus is the most sensitive to ischemia and hypoxia part of brain tissue; e.g., CIH has been shown to damage hippocampal neurons and increase the number of microglial cells in mice [13]. At the beginning of the disease, those symptoms might not be observed due to current neurocompensatory mechanisms, which, on the other hand, may impede the detection of cognitive impairment and the implementation of early intervention [14]. Thus, this review aims to summarize the literature on neuroprotective mechanisms in OSA, present the available clinical findings, and propose potential aims for new treatment.

### Search Strategy and Literature Selection

To ensure a structured, balanced, and comprehensive identification of evidence, an extensive literature search was conducted across major biomedical databases, including PubMed/MEDLINE, Embase, and Google Scholar, covering records published from database inception up to January 2026. The search strategy employed combinations of controlled vocabulary (MeSH) and free-text keywords, structured around two core conceptual pillars: (1) OSA and its hallmarks (e.g., “obstructive sleep apnea”, “OSA”, “chronic intermittent hypoxia”, “CIH”, “sleep fragmentation”, “continuous positive airway pressure” or “CPAP”), and (2) neurocognitive processes and molecular pathways (e.g., “neuroprotection”, “neurodegeneration”, “cognitive decline”, “SUMOylation”, “SENP1”, “NLRP3 inflammasome”, “Toll-like receptors”, “TLR2”, “TLR4”, “erythropoietin”, “glycolysis”, “lactate”, “BDNF”, “neurotrophins”).

The selection encompassed peer-reviewed original research articles (pre-clinical in vitro and animal CIH models, as well as human observational and interventional cohort studies) and relevant high-impact reviews. Articles were restricted to the English language. Studies were included if they directly examined the molecular, metabolic, or cellular mechanisms underlying cognitive dysfunction, neuronal resilience, or neuroprotection under intermittent hypoxia, or if they evaluated peripheral biomarkers and therapeutic interventions (e.g., CPAP) correlating with neurocognitive outcomes in OSA. Exclusion criteria comprised non-peer-reviewed conference abstracts, case reports, studies focusing exclusively on peripheral non-neural organ pathology without any neurological context, and studies where hypoxia was continuous rather than intermittent. Reference lists of eligible studies were manually cross-checked to capture additional relevant publications.

## 2. Neurobiological Action Mechanisms of CIH on Neural Tissue

As mentioned above, OSA may affect the central nervous system, leading to degenerative changes in brain function. One of the main signaling pathways includes CIH-associated transmission. Crucially, while CIH represents a primary molecular trigger, neural tissue in OSA is simultaneously subjected to sleep architecture fragmentation, intermittent hypercapnia, recurrent intrathoracic pressure swings, and autonomic surges, which may act additively or synergistically to promote neurocognitive deficits. CIH may act through neuroinflammation and nitric oxide (NO), which is involved in cellular oxidative stress [15]. Neuronal injury, indicated by, e.g., quantitative changes in the hippocampus (decrease/increase, depending on sex) or qualitative changes in the cortex (functional connectivity enhancements in specific regions) [16], results in decreased synaptic plasticity and subsequent neurocognitive decline [17]. Neuroinflammation induced by CIH in OSA is persistent and unrestricted to a particular brain structure. Additionally, it may damage the integrity of the blood–brain barrier (BBB), increasing BBB permeability and further intensifying neuroinflammation [18]. Sustained CIH activates microglia cells, which act through synaptic pruning and increase the levels of proinflammatory cytokines, oxidative and nitrosative stressors, and neurotoxic substances, such as proteases, glutamate, or chemokines. The mechanisms of action are described in the following paragraphs.

### 2.1. Enhanced Brain SUMOylation

SUMOylation, as a reversible posttranslational modification, participates in the regulation of cellular activities and protein stabilization. It works through an ATP-dependent pathway, covalently attaching to recognized lysine residues of target proteins, with the help of, e.g., the SUMO-conjugating enzyme ubiquitin-conjugating enzyme 9 (Ubc9), the best-known enzyme that is highly expressed peripherally. SUMOylation is a reversible process that involves sentrin/SUMO-specific proteases (SENP), with SENP1 being the most prominent isopeptide. SUMO/deSUMOylation balance is a crucial mechanism of coping with oxidative stress in response to CIH, restoring proper intracellular redox state. Thus, the possible mechanism of action and activity can be controlled via two contradictory molecules, depicted as the “on-switch” (the Ubc9 enzyme) and its “off-switch” (the SENP1 protease). SUMOylation is ubiquitously present in both neuronal and peripheral tissues. Its relationship with hypoxia-inducible factor 1α (HIF-1α) is bidirectional: HIF-1α can be SUMOylated, decreasing its stability, and global SUMOylation of other proteins can be enhanced by HIF-1α. In neuronal-associated pathways, in vivo and in vitro, CIH increased SUMOylation of peroxisome proliferator-activated receptor γ (PPAR-γ) in microglia and the hippocampus while simultaneously leading to downregulation of SENP1 [19]. Inactivated SUMOylated PPAR-γ did not inhibit inflammatory genes (e.g., the nuclear factor kappa-light-chain-enhancer of activated B cells (NF-κB) pathway), which may be associated with the observed cognitive decline. In general, PPAR-γ has emerged as a key regulator of inflammatory processes and immune responses in chronic inflammatory diseases, including neurodegenerative ones [20]. In addition, peripheral PPAR-γ levels have been shown to be dysregulated in neurological conditions [21], and administering its activators might alleviate neurological symptoms [21].

Besides PPAR-γ, CIH-mediated SENP1 downregulation has been shown to suppress Wnt/β-catenin signaling in rodent hippocampal microglia [22]. However, the broader downstream link, whereby hyper-SUMOylation stabilizes GSK3 and silences β-catenin to drive widespread neuroaxonal degradation, represents a mechanistic hypothesis extrapolated from related oncological and neurodegenerative systems rather than an exhaustively validated cascade in OSA neural tissue. While its neuroprotective role is clinically recognized in general neurodegenerative contexts [23], the specific involvement of SUMOylation-mediated Wnt/β-catenin suppression in OSA is currently suggested by preclinical evidence. Specifically, as demonstrated in animal and cell-culture CIH models, aberrant hyper-SUMOylation of upstream regulators stabilizes active GSK3 or impedes its inhibitory phosphorylation, accelerating β-catenin clearance. This model-derived cascade hyperactivates GSK3 and suppresses β-catenin expression, shifting the cellular equilibrium toward an anti-plasticity state that promotes neuroaxonal injury; however, direct translational confirmation in the brain tissue of human OSA patients remains to be established.

**Figure 1:**
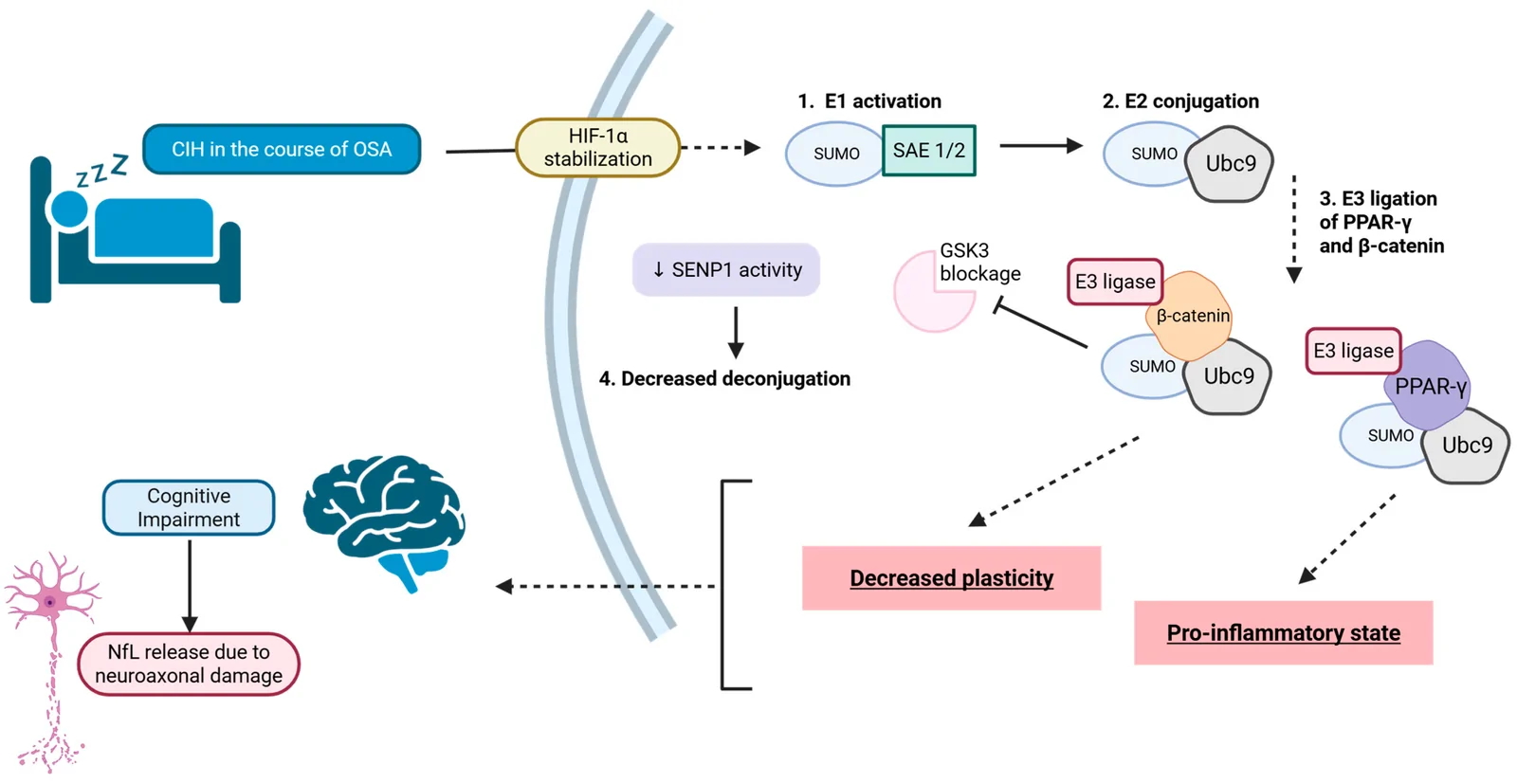
The suggested mechanism of CIH influence on SUMOylation and its consequences. CIH stabilizes HIF-1α, driving an enzymatic cascade that enhances global SUMOylation via SAE1/2 (E1 activation) and Ubc9 (E2 conjugation). CIH downregulates the deSUMOylating enzyme SENP1. Hyper-SUMOylation leads to the functional inactivation of PPAR-γ, promoting a proinflammatory microglial state, and hyperactivates GSK3, which accelerates β-catenin degradation. This impairs synaptic plasticity, induces axonal damage, triggering the release of NfL as a marker of cognitive decline. Abbreviations: CIH—chronic intermittent hypoxia, GSK3—Glycogen Synthase Kinase 3, HIF-1α—hypoxia-inducible factor 1α, NfL—Neurofilament Light, PPAR-γ—Peroxisome Proliferator-Activated Receptor γ, SAE—SUMO-activating enzyme, SENP1—SUMO-specific peptidase, SUMO—small ubiquitin-like modifier, Ubc9—SUMO-conjugating enzyme UBC9. Note: Solid arrows indicate pathways experimentally demonstrated under CIH conditions, whereas dashed arrows denote proposed relationships derived from related neurodegenerative models. The small downward arrow indicates downregulation of the molecule, and underlined text indicates final molecular alterations.

Ultimately, the SUMOylation-driven loss of synaptic plasticity and ongoing neuroaxonal injury lead to the structural degradation of neurons, which is reflected peripherally by the release of neurofilament light chain (NfL)—a sensitive biomarker of axonal degeneration—into the circulation [24]. The suggested hypothesis is depicted in Figure 1.

### 2.2. Pattern Recognition Receptor Family Dysregulation

The second signaling pathway involved in degenerative brain conditions includes the effect of multiprotein cytosolic complexes called inflammasomes, with the most promising effect of NOD-like receptor pyrin domain-containing protein 3 (NLRP3). Inflammasomes are protein complexes that assemble when recognizing pathogen-associated molecular patterns (PAMPs) or damage-associated molecular patterns (DAMPs) [25]. Their mechanism of action includes inducing inflammation through modulating caspase-1 activation. Preclinical-only evidence indicates that NLRP3 inflammasome assembly induces spatial learning and memory deficits in the rodent CIH paradigms [13], potentially driven by mitochondrial reactive oxygen species (mROS) [26]; however, direct confirmation in central human brain tissue is entirely lacking, with clinical data restricted to peripheral surrogate cells. As demonstrated in an animal model, Wu et al. reported that NLRP3 depletion ameliorated CIH-induced neuronal damage, while NLRP3 knockout additionally decreased damaged mitochondria and reduced oxidative stress levels, as indicated by malondialdehyde and superoxide dismutase [27]. Increased stress response and redox imbalance, indicated by thioredoxin-interacting protein (TXNIP) activation, may also lead to NLRP3 activation, as shown in human endothelial cells [28]. NLRP3 activation was shown to be suppressed by blockade of the cannabinoid 1 receptor (CBR1); conversely, it can be speculated that increased CBR1 activation may lead to enhanced mROS production and, in turn, to NLRP3 activation [29]. Activated NLRP3, in conjunction with the apoptosis-associated speck-like protein containing a caspase recruitment domain (ASC), activates caspase 1. It affects neuronal tissue, leading to neuroinflammation, possibly through the transformation of A1/A2 astrocytes [30]. Beyond pro-inflammatory cytokine maturation, activated Caspase-1 cleaves Gasdermin D (GSDMD) into its N-terminal fragment, which forms oligomeric pores in the plasma membrane, ultimately driving pyroptotic cell death and severe neuroinflammation. As shown in a report by Ren et al., in the hippocampus of rats exposed to CIH, inflammasome expression (NLRP3/Caspase-1/ASC/IL-1β) increased, and the transformation from A2 to A1 astrocytes was enhanced. In addition, the changes correlated with a decline in spatial learning performance. Inhibition of the NLRP3 inflammasome reduced astrocyte activation, reversed A2-to-A1 transformation, and, unexpectedly, increased Nissl bodies, which had a protective effect on the brain. Additionally, a decrease in proinflammatory agents, such as tumor necrosis factor α, IL-1β, and IL-6 [31]. Nissl bodies, which comprise endoplasmic reticulum and free ribosomes, undergo chromatolysis under CIH, and their depletion is a visible manifestation of endoplasmic reticulum stress. Prolonged endoplasmic reticulum stress in response to CIH leads to impaired synaptic plasticity and long-term memory deficits [32]. In astrocytes, under normal conditions, microRNA-223-3p promotes neuroprotection and, in pathological states, relieves neuronal damage [33]; additionally, it exhibits strong binding to NLRP3. Under CIH conditions, the increase in NLRP3 expression was associated with lower microRNA-223-3p levels, thereby attenuating neuroinflammation. Therefore, the NLRP3/microRNA-223-3p axis may affect neuronal tissue, especially astrocytes, due to the unfavorable effects of CIH [31].

**Figure 2:**
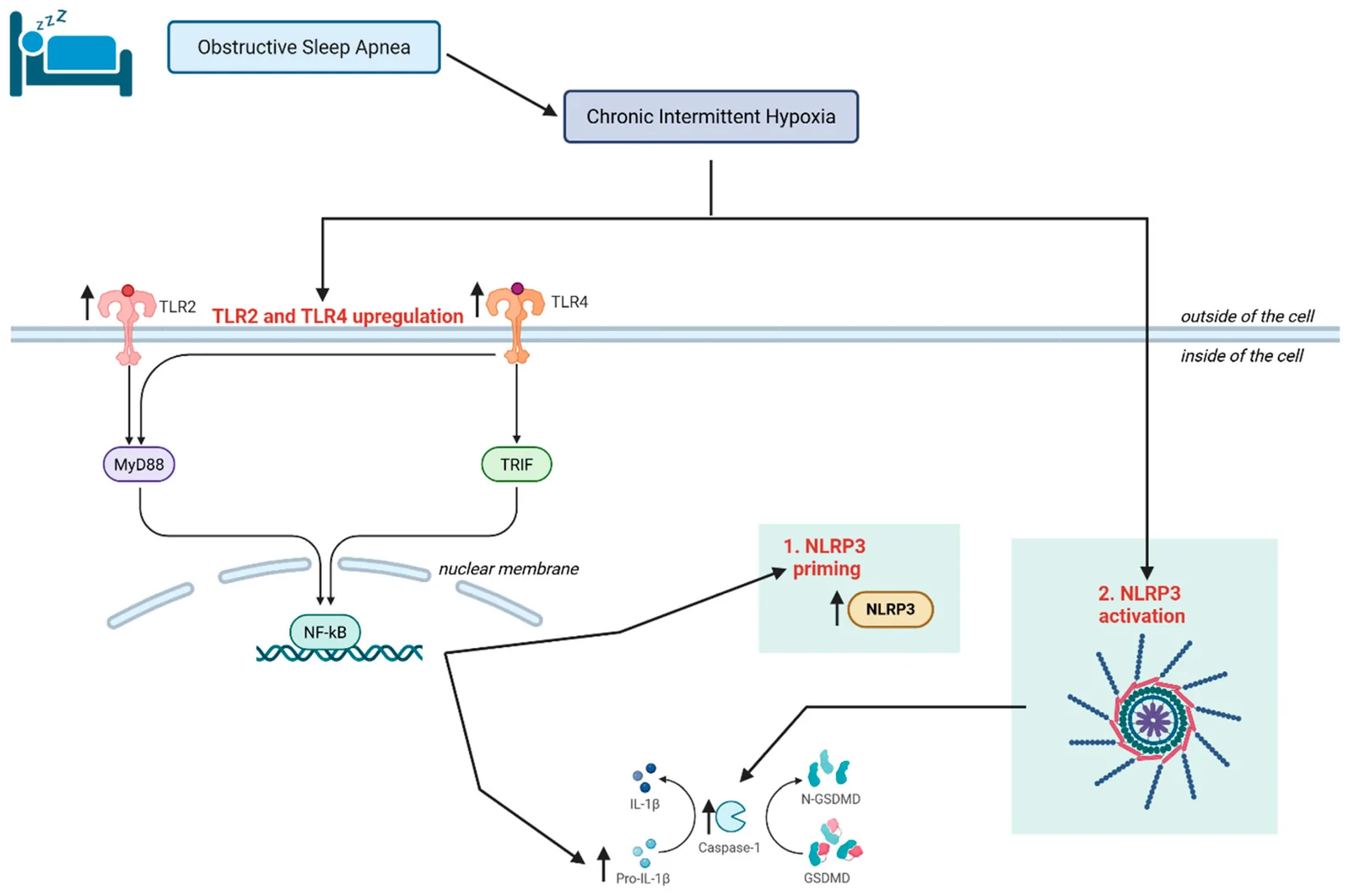
The mechanism of CIH influence on pattern recognition receptor family dysregulation. OSA-induced CIH upregulates membrane-bound TLR2 and TLR4. TLR signaling through MyD88- and TRIF-dependent pathways converges on NF-κB activation, which enters the nucleus to initiate the priming phase of NLRP3 inflammasome components and pro-inflammatory cytokines, e.g., pro-IL-1β. The second activation signal triggers assembly of the NLRP3 inflammasome complex, leading to Caspase-1 cleavage. Caspase-1 subsequently processes pro-IL-1β into active IL-1β and cleaves GSDMD into its active N-GSDMD, executing pyroptosis and propagating neuroinflammation. Abbreviations: GSDMD—Gasdermin D; IL-1β—Interleukin-1 beta; NF-kB—Nuclear Factor Kappa B; NLRP3—NLR Family Pyrin Domain Containing 3; Pro-IL-1β—Pro-interleukin-1 beta; TLR2—Toll-like Receptor 2; TLR4—Toll-like Receptor 4; TRIF—TIR Domain Containing Adapting Molecule 1 (Toll-IL-1 Receptor-domain-containing adapter-inducing interferon-beta); MyD88—Myeloid Differentiation Primary Response 88. Note: The small arrow indicates upregulation of the molecule.

Toll-like receptors (TLRs), together with NLRs, belong to the pattern recognition receptor family and are located at the cell membrane and on endosomes; their mode of action includes proinflammatory transcriptional regulation. One of the best-known TLRs associated with CIH and sterile inflammation is TLR4. The microglial TLR4 mRNA expression increased up to 7-fold in animal neuronal tissue exposed to CIH and correlated with microglial inflammatory gene expression—cyclooxygenase-2, IL-1β, IL-6, and NO synthase [34]. As TLR4 is the only one from TLRs that may act through 2 pathways—myeloid differentiation primary response 88 (MyD88) and toll/interleukin-1 receptor-domain-containing adapter-inducing interferon-β (TRIF), both of them are involved in CIH-induced damage, leading to NF-κB activation. Wanting et al. reported that atorvastatin reversed the harmful effects of CIH on the animal hippocampal tissue (via alleviation of elevated TLR4, MyD88, and TRIF) by decreasing superoxide dismutase (SOD) and malondialdehyde (MDA)—indicators of oxidative stress [35]. It supports the need for investigation of potential therapeutic options. The second TLR engaged in CIH damage may be TLR2, as research has shown that its knock-out alleviated neuroinflammation in the hippocampal tissue of mice by blocking the TLR2-MYD88 pathway. Additionally, there was an improvement in spatial learning and memorization [36]. Treating CIH-exposed mice with TLR2 antagonism—ortho-vanillin—had a similar beneficial effect on the hippocampus in neuroinflammation and oxidative stress alleviation [37]. TLR2 may affect the limbic system the most, as TLR2-induced neuroinflammation evaluated through magnetic resonance imaging (MRI) and bioluminescence imaging (BLI) was most expressed in the septal nuclei and forebrain [38]. In addition, TLR2 response was connected to brain-derived neurotrophic factor (BDNF), neuroplastin, and fibronectin-1 overactivity [38]. Altogether, TLR2 and TLR6 were found to have aberrant DNA methylation profiles in moderate to severe OSA patients, as follows: within the TLR2 promoter region, patients exhibited significantly increased DNA methylation at five specific CpG sites (#1, #2, #3, #25, and #28) and decreased methylation at site #18. Additionally, elevated methylation levels were observed at CpG sites #1 and #3 of the TLR6 gene [39]. In blood immune cells, these changes were also confirmed in untreated OSA patients, but not in those with CPAP treatment instituted [40]. Importantly, these peripheral epigenetic modifications demonstrate leukocyte alterations rather than direct functional activation of TLR signaling in the human central nervous system. Under CIH, their induction is coordinated by HIF-1α, which may point to hypoxic transcriptional adaptation of innate immune responses [41]. The described mechanisms are showed in Figure 2.

## 3. Potential Adaptive and Neuroprotective Mechanisms Associated with CIH in OSA

Hypoxia, in addition to the detrimental effects on the brain depicted above, may induce and enhance mechanisms that provide neuroprotection as a defense. The hypoxic ‘dose’ strictly determines this threshold, governed by nadir desaturation depth, cycle frequency, and duration [42]. In experimental rodent models, ‘low-to-moderate’ CIH typically involves modest desaturations (FiO_2_ 9–15%) in brief bouts that stabilize HIF-1α within an adaptive window, and low-dose CIH (10% O_2_) has even shown potential to improve memory in mild cognitive impairment among people [43]. Conversely, ‘severe’ preclinical CIH uses profound desaturations (FiO_2_ 5–6%, 30–60 cycles/h) over months, driving neuroinflammation and axonal decay. Crucially, directly mapping these rodent paradigms onto clinical OSA severity (classified by the AHI) remains challenging; experimental CIH isolates pure cyclical hypoxemia, whereas natural OSA combines hypercapnia, sleep fragmentation, and negative intrathoracic pressure. Furthermore, whether CIH triggers adaptation or injury in patients is heavily modulated by age, metabolic comorbidities, and disease chronicity. The main pathways leading to such a beneficial outcome include erythropoietin, aerobic and anaerobic glycolysis, antioxidative enzymes, and neurotrophic factors. A critical conceptual challenge in understanding CIH neurobiology lies in the HIF-1α paradox. While prolonged, severe intermittent hypoxia overactivates HIF-1α to trigger dysregulated post-translational modifications (e.g., hyper-SUMOylation) and persistent pro-inflammatory cascades, transient or low-to-moderate CIH stabilizes HIF-1α within an adaptive, physiological window. This dual action exemplifies a response: at moderate desaturation levels, HIF-1α acts as a neuroprotective regulator by driving the transcription of survival factors (EPO, BDNF, VEGF, GLUT1). However, when hypoxic stress exceeds this buffering capacity, chronic HIF-1α engagement shifts from adaptive compensatory signaling to decompensated, neurodegenerative toxicity. Understanding their specific characteristics may provide a baseline for identifying new methods to prevent and treat CIH-induced neurodegeneration. Their mechanisms of action are described below.

### 3.1. Erythropoietin Change

Erythropoietin (EPO) is a hormone produced primarily in the kidneys and liver that enhances red blood cell production. Its activity is driven by hypoxia, as decreased oxygen levels increase EPO production. This hormone reaches the bone marrow and stimulates stem cells to produce red blood cells more quickly, delivering oxygen to hypoxic tissues [44]. EPO is upregulated by HIFs, which stabilize under hypoxic conditions; when red blood cell levels normalize, this self-regulatory loop downregulates EPO levels [45]. The production of a hormone is suppressed in chronic kidney disease and has been reported to be changed in diseases and states connected with hypoxia, including incidental, continuous, and chronic. In CIH, EPO was shown to exhibit altered activity, suggesting an additional neuroprotective role, as EPO can cross the blood–brain barrier. Its influence on the nervous system includes inhibiting ROS generation, modulating neurotransmission, and reversing vasospasm. Furthermore, it promotes angiogenesis, attenuates apoptosis, mitigates inflammation, and facilitates stem cell recruitment [46]. Studies in rodents showed that brain-derived EPO (i.e., produced by neurons and astrocytes), as well as EPO administration, attenuates cognitive decline by downregulating NADPH levels and mitigating lipid oxidative degradation [47]. In particular, activation of the EPO/EPOR/JAK2 pathway may have a beneficial influence on cognition [48]. Activated JAK2 activates the JAK2/STAT3/PI3K/AKT/mTOR pathway, leading to neuroprotection through its anti-apoptotic properties [49]. In addition, EPO may counteract neuropathologic NLRP3 activation by inhibiting inflammasome activation in neuronal tissue [50].

The initial respiratory effect of EPO under CIH may be reversed by neocytolysis after reversion to normoxemia to protect against polycythemia, which explains the apparent absence of polycythemia in OSA despite increased EPO levels. Neocytolysis, hypothetically, is caused by increased ROS production from expanded mitochondria, which is activated by downregulated HIF-targeted B-cell lymphoma 2 interacting protein 3-like (BNIP3L) in reticulocytes [51]. The second concomitant pathway involves suppression of erythropoiesis by hepcidin, which is why OSA patients, despite increased EPO, do not develop regular erythrocytosis but may still benefit from EPO activity [52]. Reports on the topic in the OSA population are consistent with previously described data: EPO levels are higher in untreated OSA than in the control group, both in children [53] and adults [54]. Especially severe untreated OSA patients were shown to have dysregulated nocturnal EPO levels throughout the night, with an increase after 3.5 h of sleep; however, these elevated EPO levels normalized following 4 h of CPAP therapy [55]. Rather than an active suppression of neuroprotection, this decline simply reflects a physiological normalization of hypoxia-responsive signaling once arterial normoxemia is restored. Such changes did not only occur during the night—diurnal EPO levels among OSA patients resulted in lower EPO levels in response to CPAP [56] when compared to untreated ones. The second treatment modality, the mandibular advancement device, in OSA animal models statistically significantly downregulated EPO levels, along with measured HIF-1α; the effect in human samples has not been studied [57]. Interestingly, the exogenous EPO mRNA administration in the OSA mouse model diminished cognitive deficits, but without a significant change in ROS levels [58]. It may be a reflection of short-term neuroprotection ensured by EPO, but without a long-lasting change, which should be investigated in human samples.

### 3.2. Anaerobic and Aerobic Glycolysis

A metabolic shift occurs during the course of OSA, shifting toward anaerobic glycolysis as sleep-time saturation drops due to oxygen deficiency. It may be depicted by an increase in lactate, leading to mild metabolic acidosis. Excessive daytime sleepiness in OSA is inherently multifactorial, arising predominantly from severe sleep architecture fragmentation, frequent micro-arousals, and recurrent hypoxemia rather than peripheral metabolic shifts alone. However, this mechanism can also be considered as a sophisticated neuron-survival strategy. Thus, there is a fine line between toxicity and protection—what at the beginning seems to be adaptation, during a chronic condition becomes a pathological state, which points to a hormesis thesis. HIFs, regulated by prolyl-4-hydroxylase domain proteins that hydroxylate their subunits, take part in the protective hypoxia response [59]. In Ratan et al., both increased erythropoietin expression and glycolytic enzymes, together with increased HIFs, exerted a protective effect against oxidative stress-induced neuronal apoptosis, highlighting the importance of those two neuroprotective mechanisms [60]. As an essential arm of this protective hypoxic adaptation, HIF-1α stabilization upregulates key downstream target genes, including glucose transporter 1 (GLUT1), vascular endothelial growth factor (VEGF), and glycolytic enzymes [61,62,63]. Increased GLUT1 expression enhances neuronal and glial glucose uptake, while glycolytic enzymes accelerate the conversion of pyruvate to lactate to produce ATP under low-oxygen conditions. Simultaneously, VEGF induction stimulates local microvascular remodeling and angiogenesis, collectively preserving metabolic homeostasis and supporting neuronal survival during adaptive low-dose CIH.

Lactate has been shown to serve as a metabolic substrate for neurons, delivering energy more quickly than glucose when oxygen is limited. An increase in anaerobic glycolysis also enhances HIF-1α and supports neuronal survival during adaptive low-dose CIH via vasculogenesis and heat shock protein production [64]. That is why neuronal tissue, despite the general degenerative effect of such a metabolic shift, might benefit from increased lactate and survive more efficiently; however, such neuroprotection may not be sufficient in severe OSA cases. It is crucial to note that the boundary between the neuroprotective and neurotoxic effects of this metabolic shift is remarkably narrow. This suggests that the chronic imposition of anaerobic metabolism—typical of CIH in OSA—may not only deplete energy reserves but may also share overlapping metabolic cascades with the early stages of Alzheimer’s disease; however, shared metabolic vulnerabilities do not imply an identical primary etiology. In early Alzheimer’s disease models, Aβ-activated microglia, possibly due to abnormal glucose metabolism [65], might enhance anaerobic glycolysis, leading to activation of the AKT-mTOR-HIF-1α pathway, which results in impaired synaptic plasticity and Aβ aggregation [66]. Therefore, the two-sided effects of anaerobic glycolysis and its products need to be studied in OSA patients. The exact triggering factor in this group of patients might be increased NO levels [67], leading to activation of the PI3K/Akt/mTOR pathway and thus maintaining high glycolytic activity in astrocytes [68]. At the same time, under hypoxic conditions, NO inhibits HIF-1α protein accumulation [69]. This may demonstrate a modulatory effect of NO on cellular responses to hypoxic conditions.

Another adaptive pathway suggested by pre-clinical evidence involves metabolic support via acetate. As demonstrated in an animal CIH model by He et al., hypothalamic astrocytes exhibited elevated acetate levels, and exogenous acetate supplementation mitigated cognitive deficits by stimulating pyruvate carboxylase activity and restoring astrocytic glycolysis [70]. Nevertheless, this compensatory metabolic axis has been shown solely in rodent models and lacks direct validation in clinical OSA cohorts.

### 3.3. Neurotrophic Factors

Neurotrophic factors, such as the previously mentioned BDNF and its family members: nerve growth factor (NGF) and neurotrophins (NTF), are proteins involved in the survival, development, and function of neurons, acting by binding to tyrosine kinases. In OSA, their activity is regulated by CIH and oxidative stress [17]. Their protective response is significant for synaptic plasticity, neurogenesis, and neuroregeneration, whereas their disruption might reveal a degenerative influence of OSA burden on neurobehavioral outcomes.

The best-known neurotrophic factor is BDNF, which acts through the tyrosine kinase receptor B, and its decrease has been associated with reduced synaptic plasticity and increased neuronal apoptosis, leading to neurocognitive dysfunction [17]. However, studies have shown that BDNF upregulation may have a protective effect on neuronal tissue. In Yung et al., exogenous BDNF injection restored late-phase long-term potentiation in the hippocampus, suggesting potential therapeutic properties [71]. In addition, available data suggest that HIF-1α may be involved in the pathway activated by proBDNF, which induces compensatory properties in response to hypoxia in OSA patients [72]. In addition, OSA patients without clinical insomnia exhibited elevated levels of BDNF and NTF3 relative to the control group [73]. Among the OSA group, BDNF expression was positively correlated with circadian rhythm protein expression and HIF-1β, pointing to a potential relationship between neurotrophic factors and circadian changes under the influence of CIH [74]. Furthermore, in a study by Zsuga et al., OSA patients with excessive daytime sleepiness exhibited higher levels of irisin and its inducible molecule BDNF [75]. This suggests that the failure of compensatory mechanisms may stem from the chronic overexpression of these neuromodulators, rather than a deficiency in their production. Subsequent deficiency of BDNF-dependent synaptic proteins may be a key contributing factor to decompensated cognitive conditions in OSA patients [76]. Although circulating BDNF cannot be assumed to function as an uncritical proxy for central intra-cerebral concentrations due to peripheral platelet release and systemic clearance, Ricci et al. reported that OSA patients with higher peripheral BDNF levels presented with lower neurocognitive impairment [77]. What is worth mentioning: CPAP treatment was shown to decrease circulating BDNF concentrations, but not to decrease BDNF secretion, which may point to increased neuronal demand for BDNF during treatment [78]. Interestingly, although a decrease in BDNF after CPAP treatment was reported, NTF3 increased [79]. This may be explained by a compensatory mechanism in response to a decrease in BDNF, as they cooperate [80]. Molecularly, in an animal model of OSA, CIH enhanced hippocampal BDNF expression with antidepressant-like effects and increased cell proliferation [81]. This points to a two-sided relationship between CIH and the development of neurocognitive functions, as milder levels of oxygen desaturation were associated with potential beneficial effects, whereas severe deoxygenation events were associated with adverse effects. Additionally, those compensatory mechanisms might become depleted after the early phase of the disease.

**Figure 3:**
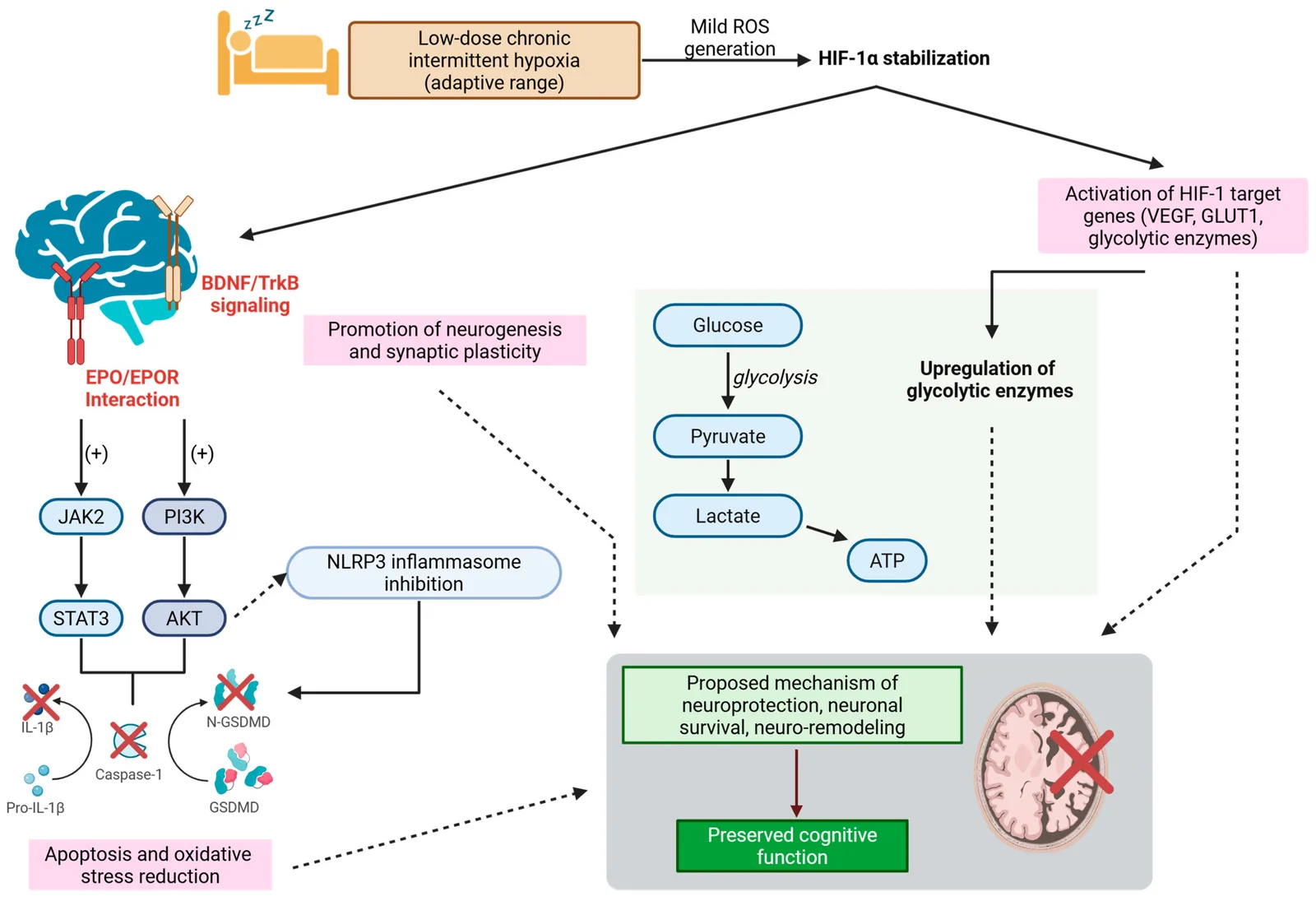
Selected adaptive mechanisms induced by low to moderate CIH in OSA. Mild ROS generation stabilizes HIF-1α, initiating two main compensatory neuroprotective axes. First, HIF-1α upregulates BDNF/TrkB and EPO/EPOR signaling, activating downstream JAK2/STAT3 and PI3K/AKT survival cascades. These pathways inhibit the NLRP3 inflammasome, suppress caspase-1-mediated apoptosis/pyroptosis, and promote neurogenesis and synaptic plasticity. Second, HIF-1α transcriptional target genes (VEGF, GLUT1, and glycolytic enzymes) facilitate glucose transport and accelerate anaerobic glycolysis (converting glucose to pyruvate and lactate to ATP). Together, these mechanisms reduce oxidative stress, preserve metabolic homeostasis, and sustain cognitive function. Abbreviations: AKT—protein kinase B; ATP—adenosine triphosphate; BDNF—brain-derived neurotrophic factor; EPO—erythropoietin; EPOR—erythropoietin receptor; GLUT1—glucose transporter 1; GSDMD—gasdermin D; HIF-1α—hypoxia-inducible factor-1 alpha; IL-1β—interleukin-1 beta; JAK2—Janus kinase 2; NLRP3—NOD-, LRR- and pyrin domain-containing protein 3; OSA—obstructive sleep apnea; PI3K—phosphoinositide 3-kinase; ROS—reactive oxygen species; STAT3—signal transducer and activator of transcription 3; TrkB—tropomyosin receptor kinase B; VEGF—vascular endothelial growth factor. Note: The “x” symbol indicates the inhibition of caspase-1, preventing the maturation of IL-1β and cleavage of GSDMD into N-GSDMD. Pathways shown represent established pre-clinical animal/in vitro cascades; translation to human central neurobiology remains under ongoing investigation; solid arrows indicate experimentally validated signaling pathways in pre-clinical models; dashed arrows indicate proposed, hypothesized, or multi-step functional connections.

Besides BDNF, other family factors such as NGF and NTFs have also been studied, however, not in as much detail. Fang et al. observed that under CIH, there was a statistically significant upregulation of NGF, together with other RAS signaling molecules (EGF and FYB1) [82]. Similarly, NGF upregulation was present among the pediatric OSA group [83]. The presented mechanism is depicted in Figure 3.

## 4. Clinical Significance, Limitations and Further Research Perspectives

### 4.1. Clinical Significance

When translating basic mechanistic insights into clinical practice, several inherent limitations of animal models must be acknowledged. Standard rodent CIH protocols primarily mimic cyclic arterial oxygen desaturation, but fail to fully capture the complex, multi-systemic pathophysiology of human OSA. Unlike experimental CIH, clinical OSA is characterized by recurrent hypercapnia, intrathoracic pressure swings due to upper airway resistance, sleep architecture fragmentation (arousal index), and chronic autonomic instability. Moreover, animal models typically isolate young, healthy rodents without the confounding metabolic and cardiovascular comorbidities (e.g., obesity, hypertension, diabetes) commonly present in the human OSA population. Thus, future translational studies must integrate multi-factorial models to better reflect human sleep-disordered breathing.

An important implication of the mechanisms discussed in this review is that future management of OSA-associated cognitive impairment may require a shift from a uniform treatment approach toward precision medicine strategies. Although continuous positive airway pressure effectively eliminates upper airway obstruction and intermittent hypoxia, substantial interindividual variability in neurocognitive outcomes persists despite adequate treatment adherence. This observation suggests that the capacity to activate endogenous neuroprotective pathways may differ among patients and could influence long-term neurological resilience. However, this variability cannot be attributed solely to the depletion of endogenous neuroprotective mechanisms; it is also heavily confounded by non-molecular determinants [84] including daily treatment adherence, duration of therapy, baseline neurocognitive status, advanced age, pre-existing irreversible neural injury before CPAP initiation, clinical OSA phenotypes (e.g., REM-related vs. NREM-related), vascular and metabolic comorbidities, and methodological heterogeneity across cognitive assessment tools. Consequently, combining conventional OSA therapy with molecular biomarkers reflecting both neurodegenerative burden and neuroprotective reserve may improve patient stratification, facilitate earlier identification of individuals at increased risk of cognitive decline, and support the development of targeted adjunctive therapies. The use of brain penetration technologies and targeted central nervous system drug delivery might resolve the hurdle of translating molecular neuroprotection into effective therapies for OSA-induced neurocognitive decline due to the selective BBB permeability [85]. Because peripheral delivery of neuroprotective agents often yields limited central bioavailability, integrating advanced, targeted brain-penetration technologies holds significant therapeutic promise. Strategies such as intranasal delivery [86], engineered polymeric nanoparticles [87], and focused ultrasound-mediated transient BBB opening [88] could enable therapeutic concentrations of neuroprotective agents to reach vulnerable subcortical regions like the hippocampus. Developing and combining these delivery platforms with OSA-specific treatment might represent a promising frontier for future translational neurobiology and neurorehabilitation studies.

Synthesizing the preclinical and observational evidence, we introduce the term ‘neuroprotective reserve’ as a novel, hypothesis-generating conceptual framework newly formulated in this review. Drawing conceptual inspiration from classical models of cognitive and brain reserve, we define this construct as the brain’s intrinsic capacity to counterbalance CIH-induced pathways involving HIF-1α, erythropoietin, neurotrophic factors, astrocytic metabolic change, and antioxidant defense. Rather than representing an established, clinically validated metric, this construct serves as a theoretical paradigm to conceptualize how endogenous resilience operates as an active biological buffer. As disease severity and chronicity increase, these compensatory mechanisms may become progressively exhausted or maladaptive, shifting the balance toward persistent neuroinflammation, synaptic dysfunction, and cognitive decline. Prospective translational and longitudinal clinical studies will be essential to validate, quantify, and operationalize this construct through multi-modal panels of circulating biomarkers and advanced neuroimaging.

### 4.2. Limitations

Several inherent methodological limitations of this narrative review must be acknowledged. First, the narrative synthesis carries a potential risk of selection bias relative to systematic reviews. Second, significant heterogeneity exists across experimental CIH protocols regarding nadir depth, cycle frequency, and duration, which hampers direct cross-study comparability. Third, in human cohorts, definitions of OSA severity rely heavily on the AHI, which fails to capture hypoxic burden nuances. Finally, clinical studies suffer from varied neurocognitive testing batteries, heavy reliance on circulating peripheral biomarkers as speculative proxies for central nervous system processes, and an absence of prospective, longitudinal cohort data tracking molecular shifts over time.

A significant limitation of the hypothetical neuroprotective mechanisms described in this article seems to be their restricted effect—only in normal brain tissue. Although the neuroprotective reserve provides a temporary buffer against CIH, this delicate compensation remains highly vulnerable to superimposed acute vascular events, most notably ischemic stroke [89]. Under acute cerebral ischemia, intrinsic adaptive pathways are rapidly overwhelmed. The catastrophic collapse of tissue perfusion induces severe bioenergetic failure in the form of rapid ATP depletion [90] and extensive BBB disruption [91]. In this decompensated setting, suggested physiological adaptations such as HIF-1α-driven anaerobic glycolysis can no longer sustain neuronal viability; instead, profound intracellular lactic acidosis and massive mitochondrial reactive oxygen species may trigger microglial hyperactivation, accelerated caspase-1/GSDMD cleavage, and penumbral pyroptosis. The immediate outcome of this resilience destruction is a rapid loss of structural and functional connectivity, manifesting as acute neuroaxonal degradation and aggravated neurological deficits. Over the long term, OSA-related neuroinflammation and hyper-SUMOylation synergize with post-ischemic cascades, driving chronic white matter rarefaction, impaired hippocampal synaptic plasticity, and markedly elevated susceptibility to post-stroke cognitive impairment and secondary vascular dementia [89].

### 4.3. Further Research Perspectives

The described neurodegenerative and neuroprotective mechanisms provide a framework for identifying potential therapeutic targets in the prevention and treatment of cognitive function impairment, in particular in OSA patients, who are at high risk of developing neurocognitive decline due to the burden of the disorder. This is supported by the higher prevalence of Alzheimer’s disease [92] and Parkinson’s disease [93] in the OSA population, suggesting that the current clinical approach should focus on preventing or delaying such neurodegenerative changes, which lead to cognitive function decline. Future studies should consider both blocking neurodegenerative pathways and enhancing neuroprotective signaling. In addition, these mechanisms of action should be evaluated in the context of the presence or absence of OSA treatment, such as CPAP, with particular attention to adequate compliance. Additionally, the development of specific biomarkers that could enable rapid and inexpensive monitoring of changes in cognitive function is needed for the OSA population. It would be particularly useful to identify biomarkers associated with oxygen metabolism that are specific to hypoxia-associated conditions. A low number of high-quality reports evaluating neurocognitive function and underlying mechanisms in OSA patients limits possible extrapolation of the data to a larger OSA population. Furthermore, studies should consider confounding factors such as OSA phenotypes [94,95,96] and comorbidities [73,97,98] that are likely to affect the development and progression of cognitive decline. Studies that bridge the gap between animal models and clinical practice are needed. Moreover, future research should utilize advanced neuroimaging techniques, such as Magnetic Resonance Spectroscopy, to non-invasively quantify cerebral lactate concentrations and metabolic shifts in OSA patients during hypoxic events. All of the above may contribute to a more personalized medical approach and a multidimensional assessment of the patient. To facilitate the distinction between pre-clinical animal models and human OSA cohorts, key findings and the corresponding levels of evidence are summarized in Table 1.

## 5. Conclusions

OSA represents a significant twenty-first-century health challenge, evolving into a systemic multifactorial disorder, whose burdens extend far beyond respiratory dysfunction. The cornerstone of its neurological impact is CIH, which drives a complex interplay between neurodegenerative damage and endogenous neuroprotective responses. To better contextualize these findings, three domains must be distinguished.

(a) Current Clinical Management of OSA

The clinical management of OSA may ultimately benefit from a more multidimensional and personalized approach. While CPAP remains the established first-line therapy, it may not fully address the established molecular imbalances, warranting the exploration of potential investigational approaches, such as exogenous antioxidants or pharmacological modulators of neuroprotective pathways. Thus, an understanding of such mechanisms may help identify high-risk patients, who, despite good compliance with CPAP, still exhibit cognitive deficits.

(b) Candidate Mechanisms and Biomarkers

Mechanistic studies, primarily demonstrated in animal CIH models, indicate that hypoxic neuronal injury is driven by persistent neuroinflammation and counteracts neuroprotective agents; while supported by human observational data in peripheral tissues, the extent to which these specific molecular axes drive central neurocognitive decline in clinical OSA requires rigorous clinical validation. These pathways collectively contribute to oxidative stress, synaptic pruning, and cognitive decline, particularly in the hippocampus’s sensitive tissues. Simultaneously, the brain initiates several physiological defense mechanisms to counteract these detrimental effects.

(c) Hypothetical and Pre-clinical Therapeutic Target

Ultimately, bridging the gap between molecular findings and clinical interventions is essential to preventing irreversible neurodegenerative outcomes in the growing OSA population. The findings summarized in this review suggest that the neurological consequences of OSA are determined not only by the cumulative burden of CIH but also by the individual’s neuroprotective reserve, namely the capacity of endogenous adaptive mechanisms to preserve neuronal integrity despite repeated hypoxic insults.

## Figures and Tables

**Table 1:** The comparison of confirmed changes among animal models vs. OSA patients. Abbreviations: BDNF—Brain-Derived Neurotrophic Factor; CIH—Chronic Intermittent Hypoxia; CPAP—Continuous Positive Airway Pressure; DNA—Deoxyribonucleic Acid; EPO—Erythropoietin; mRNA—Messenger Ribonucleic Acid; NLRP3—NLR Family Pyrin Domain Containing 3; NTF3—Neurotrophin-3; OSA—Obstructive Sleep Apnea; SENP1—Sentrin-Specific Protease 1; SUMO—Small Ubiquitin-like Modifier; TLR2—Toll-like Receptor 2; TLR4—Toll-like Receptor 4; TLR6—Toll-like Receptor 6; TXNIP—Thioredoxin Interacting Protein.

Observed Change	Animal Model	OSA Patients	References	Kind of Evidence	Target Tissue in Humans
Enhanced brain SUMOylation	SENP1 downregulation leads to apoptosis via pathways depicted in Figure 1; its overexpression promotes M2 microglial activation and inhibits inflammatory genes	Lack of direct confirmation; currently identified as a speculative target for therapeutic intervention	[19,22]	Animal CIH model, in vitro cell culture	Not confirmed
Inflammasomes overactivity	CIH induces spatial learning decline and memory impairment via NLRP3 activation; NLRP3 knockout reduces damaged mitochondria and oxidative stress	Lack of direct confirmation; though TXNIP-induced redox imbalance (a potential NLRP3 activator) has been noted in human endothelial cells	[13,27,28,31]	Animal CIH model, in vitro human endothelial cells	Endothelial cells
Toll-like receptors upregulation	TLR4 mRNA increases up to 7-fold in microglial tissue under CIH; TLR2 knockout or antagonism alleviates neuroinflammation and improves spatial learning	OSA patients exhibit aberrant DNA methylation profiles in TLR2 and TLR6 genes; these changes are reversed or absent in patients treated with CPAP	[34,35,36,37,38,39]	Animal CIH model, human observational study	Peripheral blood leukocytes
Erythropoietin change	Brain-derived EPO or exogenous administration attenuates cognitive decline by mitigating lipid oxidative degradation	EPO levels are consistently higher in both pediatric and adult OSA patients compared to controls; nocturnal and diurnal levels normalize in response to CPAP	[45,47,48,50,52,53,54,55,56,58]	Animal CIH model, human observational and interventional cohort studies	Serum, plasma
Anaerobic or aerobic glycolysis	Acetate infusion restores glycolysis in hypothalamic astrocytes and mitigates cognitive impairment in OSA mouse models	Lack of direct confirmation; no studies concern this specific topic in human OSA samples	[60,64,68,69]	Animal CIH model, in vitro astroglial assays	Not confirmed
Neurotrophic factors	Exogenous BDNF restores late-phase long-term potentiation in the hippocampus; CIH-enhanced BDNF may have antidepressant-like effects	High peripheral BDNF levels correlate with lower neurocognitive impairment; CPAP treatment significantly alters circulating BDNF and NTF3 concentrations	[72,73,74,78,79]	Animal CIH model, human observational and interventional cohort studies	Serum, plasma

## Data Availability

No new data were created or analyzed in this study. Data sharing is not applicable to this article.

## Abbreviations

The following abbreviations are used in this manuscript:

Abbreviation	Definition
AKT	protein kinase B
ASC	apoptosis-associated speck-like protein containing a caspase recruitment domain
ATP	adenosine triphosphate
BBB	blood-brain barrier
BDNF	brain-derived neurotrophic factor
BLI	bioluminescence imaging
BNIP3L	B-cell lymphoma 2 interacting protein 3-like
CBR1	cannabinoid 1 receptor
CIH	chronic intermittent hypoxia
CPAP	continuous positive airway pressure
DAMPs	damage-associated molecular patterns
DNA	deoxyribonucleic acid
EPO	erythropoietin
EPOR	erythropoietin receptor
GLUT1	glucose transporter 1
GSDMD	gasdermin D
GSK3	glycogen synthase kinase 3
HIF-1α	hypoxia-inducible factor-1 alpha
IL-1β	interleukin-1 beta
JAK2	janus kinase 2
MDA	malondialdehyde
MRI	magnetic resonance imaging
mRNA	messenger ribonucleic acid
mROS	mitochondrial reactive oxygen species
MyD88	myeloid differentiation primary response 88
NF-κB	nuclear factor kappa-light-chain-enhancer of activated B cells
NfL	neurofilament light chain
NGF	nerve growth factor
NLRP3	NOD-like receptor pyrin domain-containing protein 3
NO	nitric oxide
NTF	neurotrophins
NTF3	neurotrophin-3
OSA	obstructive sleep apnea
PAMPs	pathogen-associated molecular patterns
PI3K	phosphoinositide 3-kinase
PPAR-γ	peroxisome proliferator-activated receptor γ
PSG	polysomnography
ROS	reactive oxygen species
SAE	SUMO-activating enzyme
SENP	sentrin/SUMO-specific proteases
SENP1	SUMO-specific peptidase
SOD	superoxide dismutase
STAT3	signal transducer and activator of transcription 3
SUMO	small ubiquitin-like modifier
TLRs	toll-like receptors
TLR2	toll-like receptor 2
TLR4	toll-like receptor 4
TLR6	toll-like receptor 6
TRIF	toll/interleukin-1 receptor-domain-containing adapter-inducing interferon-β
TrkB	tropomyosin receptor kinase B
TXNIP	thioredoxin-interacting protein
Ubc9	ubiquitin-conjugating enzyme 9
VEGF	vascular endothelial growth factor
